# Palliation of chronic breathlessness with morphine in patients with fibrotic interstitial lung disease – a randomised placebo-controlled trial

**DOI:** 10.1186/s12931-020-01452-7

**Published:** 2020-07-23

**Authors:** Sissel Kronborg-White, Charlotte Uggerhøj Andersen, Charlotte Kohberg, Ole Hilberg, Elisabeth Bendstrup

**Affiliations:** 1 Department of Respiratory Diseases and Allergy, Aarhus University Hospital, 8200 Aarhus N, Denmark; 2 Department of Internal Medicine, Viborg Regional Hospital, 8800 Viborg, Denmark; 3 Department of Pharmacology, Aarhus University Hospital, 8200 Aarhus N, Denmark; 4 Department of Oncology, Odense University Hospital, Odense, 5000 Denmark; 5 Department of Internal Medicine, Lillebaelt Hospital, 7100 Vejle, Denmark

**Keywords:** Interstitial lung disease, Palliation, Morphine

## Abstract

**Background:**

Patients suffering from fibrotic interstitial lung diseases (fILD) have a poor prognosis and a high symptom burden. Palliative treatment includes relief of symptoms such as breathlessness. There is no evidence-based treatment for chronic breathlessness but opioids are often used despite concerns due to the hypothetical risk of respiratory depression. This study investigated the effect of oral morphine drops in patients with fILD on chronic breathlessness and safety.

**Methods:**

In a double-blinded placebo-controlled study, 36 patients with fILD were randomised to either four daily doses of 5 mg of oral morphine drops or placebo for 1 week. Endpoints and safety parameters were obtained at baseline, at follow-up after 1 h and 1 week.

**Results:**

The primary endpoint, the visual analogue score (VAS) of dyspnea was reduced by 1.1 ± 0.33 cm in the morphine group at follow-up compared to baseline (*P* < 0.01), whereas the reduction was 0.35 ± 0.47 cm in the placebo group. However, the difference between the two groups was not statistically significant (*p* = 0.2). Oral morphine drops did not affect respiratory frequency, pulse rate, blood pressure, peripheral saturation or the 6-min walk test. More patients treated with morphine reported constipation, nausea and confusion.

**Conclusion:**

Oral administration of morphine drops, 20 mg a day, in patients with fILD did not significantly reduce dyspnea VAS score during 1 week compared to placebo. Oral morphine did not induce respiratory depression, but was related to an increased risk of constipation, nausea and confusion.

**Trial registration:**

The trial is registered in clinicaltrials.gov (Identifier: NCT02622022). Registered 4 December 2015.

## Background

Fibrotic interstitial lung diseases (fILD) form a group of serious lung scarring diseases with a poor prognosis [1]. Idiopathic pulmonary fibrosis (IPF) is the most frequent type and is an irreversible progressive and fatal interstitial lung disease with an average survival of three to 5 years after diagnosis. Other types of fILD including chronic hypersensitivity pneumonitis and connective tissue-related interstitial lung diseases may also show a progressive phenotype with compromised survival [2]. Despite an increasing number of randomised clinical trials over the past 10–15 year, there are no curative treatments although new anti-fibrotic drugs have recently been shown to slow progression and prolong survival in patients with mild to moderate IPF [3–5].

Despite the optimism caused by new antifibrotic treatments, a considerable number of patients with fILD will either not fulfil the criteria for treatment or tolerate the drugs due to side effects. Moreover, most patients will inevitably experience disease progression and eventually need palliative treatment. Chronic breathlessness is a highly prevalent symptom in fILD and symptom relief is an important aspect of palliative care in these patients [6]. Most patients with fILD will experience progressive breathlessness affecting their quality of life and some will suffer from excruciating shortness of breath in the terminal stage of disease [7]. There is no evidence-based treatment for chronic breathlessness, but opioids are often used in daily clinical practice. Due to the well-known respiratory depressant effect of opioids, treatment is often used reluctantly by health care professionals due to fear of reducing oxygenation [8].

To our knowledge, there has only been one single interventional study showing that a subcutaneously administered single-dose of low-dose diamorphine alleviated shortness of breath without affecting respiration in patients with fILD [9]. Two recent retrospective, non-controlled studies in terminally ill patients with fILD reported reduced dyspnea during subcutaneous morphine administration without significant depression of the respiratory rate [8, 10].

Thus, there is lack of high-quality, prospective randomised studies for treatment of chronic breathlessness in fILD. The aim of the present study was to investigate the effect on chronic breathlessness and quality of life as well as safety of oral administration of morphine drops for 1 week in patients with fILD.

## Methods

### Study design

The study was a double-blinded randomised placebo-controlled study. Randomisation to morphine treatment or placebo in 1:1 ratio, was undertaken electronically by the pharmacy that produced the study medication following the rules of good manufacturing practice. The study medication consisted of magistral oral morphine drops 20 mg/ml (morphine) and identical bottles containing the same constituents except for morphine (placebo). Patients were treated for 7 days with five drops of morphine corresponding to 5 mg four times a day. The participants were allowed five drops extra as needed maximally four times a day. All patients were offered laxatives and recommended to start treatment if constipated.

Compliance and use of p.n. medication were controlled by medication diaries completed by the participants. The Danish Health Authority and the Central Denmark Region Committee on Health Research Ethics and the Danish Data Protection Agency approved the study protocol. The trial is registered in clinicaltrials.gov (Identifier: NCT02622022), and was monitored by the Good Clinical Practice Unit at Aarhus and Aalborg University Hospitals, Denmark.

### Study participants

Participants were recruited among patients with fILD attending the outpatient clinic at the Centre of Rare Lung Diseases, Department of Respiratory Diseases and Allergy at Aarhus University Hospital.

Patients were eligible for inclusion if they had a diagnosis of fILD according to ATS/ERS guidelines based on an overall assessment of high-resolution computer tomography (HRCT) scan, lung function tests, bronchoscopy and biopsy, if available [11], dyspnea according to the Medical Research Council (MRC) score ≥ 3, were 18 years or above and had signed an informed consent. Patients were excluded if they had an ongoing infection, reduced lung function to such an extent that any worsening of the condition could be life threatening based on the discretion of the investigator, allergic to morphine or morphine analogues, regular treatment with morphine or other opioids or if other reason for chronic breathlessness was suspected (e.g. heart failure).

### Endpoints

**Fig. 1:**
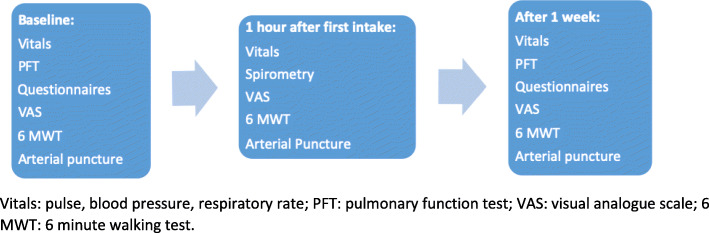
Flow diagram of the study. Vitals: pulse, blood pressure, respiratory rate; PFT: pulmonary function test; VAS: visual analogue scale; 6 MWT: 6 min walking test

The primary endpoint was patient-reported change using a visual analogue score (VAS) of dyspnea during the previous week from baseline to follow-up. For measurement of the primary endpoint, patients indicated the extent to which they had been suffering from dyspnea during the past week on a 10 cm long line from 0 to 10 [12]. Secondary endpoints were change from baseline to 1 h after the first dose of medication, and to follow up on the following parameters: respiratory rate, heart rate, systolic blood pressure, arterial partial oxygen pressure (PaO_2_), peripheral saturation at rest, six-minute walk test (6MWT) distance, and desaturation during the 6MWT. The Borg dyspnea scale was used before and after the 6MWT, a VAS score of dyspnea and a VAS score of cough during the last hour as well as and forced vital capacity (FVC) were measured. Also, change in VAS score from baseline to follow up of cough during the last week, the Leicester cough score, the King’s Brief Interstitial Lung Disease health status questionnaire (KBILD) and the Generalized Anxiety Disorder-7 questionnaire (GAD-7) were evaluated (Fig. 1).

For registration of adverse effects, participants were asked at baseline and at follow-up if they experienced constipation, nausea, headache, dizziness or confusion. They were also asked about other symptoms, which they felt were relevant at baseline and about the presence of other adverse effects at follow-up.

### Statistics

A previous study showed that a change in VAS dyspnea score of 2.1 cm was clinically relevant [12]. Under the assumption of a change of 0 cm in the placebo group and a change of 2.1 cm in the morphine group, and a standard deviation of 2.1 cm of the change in each group, and 17 patients in each group were needed to detect a 2.1 cm difference between groups with a statistical power of 80% and a significance level of 0.05. Thus, we decided to recruit 18 patients in each group.

Results from parametric data are expressed as means ± standard error, and non-parametric data are expressed as medians with interquartile ranges in brackets. Changes in endpoints from baseline to an hour after administration of medication or placebo and follow-up, respectively, were compared between groups using the unpaired t-test for parametric data and the rank sum test for non-parametric data. Changes in variables from baseline to an hour after first dose of medication or follow-up were analysed within groups using the paired t-test for parametric data and the signed rank test for non-parametric data. Changes in the proportion of patients that experienced adverse effects were compared between groups using a proportion test.

## Results

### Patients

The participants had a median age of 75 [69–78] years; 30 males and six females. There were no significant differences in baseline parameters although diffusion capacity (DLCO) in % of expected tended to be lower in the placebo group. Baseline data are presented in Table 1. None of the patients were treated with benzodiazepines for dyspnea, but 4 of the patients were treated with Zopiclon due to sleeping difficulties.

**Table 1:** Baseline demographic characteristics of participants

	Morphine group	Placebo group
Age (years)	72.5 [69.8–79.8]	75.2 [72.7–77.8]
Female (%)	16.6	16.6
BMI	28.8 ± 4.9	27.8 ± 3.7
FVC baseline (%)	73.5 ± 4.8	77 ± 6.2
DLCO baseline %	38.5 [28–50]	29.5 [26–39]
LTOT, No (%)	7 (39)	3 (6)
Diagnosis		
Idiopathic Pulmonary Fibrosis	8	9
Chronic Hypersensitivity Pneumonitis	1	3
Connective tissue disease - ILD	4	4
Unclassifiable ILD	2	1
Idiopathic non-specific interstitial pneumonia	2	1
Sarcoidosis	1	0

*BMI* body mass index, *FVC* forced vital capacity, *DLCO* diffusing capacity for carbon monoxide, *LTOT* long term oxygen therapy, *ILD* interstitial lung disease

### Compliance

Most of the participants (31) were compliant with taking the study medicine (defined as more than 90% of the dosages). Two patients from the intervention group and one patient from the placebo group forgot more than 10% of the dosages. One patient in the intervention group discontinued taking the medication due to confusion and nausea, and one patient in the placebo group discontinued taking the medication due to worsening of respiratory symptoms. The patient in the placebo group, however, completed the follow-up visit. Only one of the patients in the morphine group and three in the placebo group took extra morphine dosages when they felt the need. On average, patients took 7.25 extra dosages during the study period.

### Endpoints

**Fig. 2:**
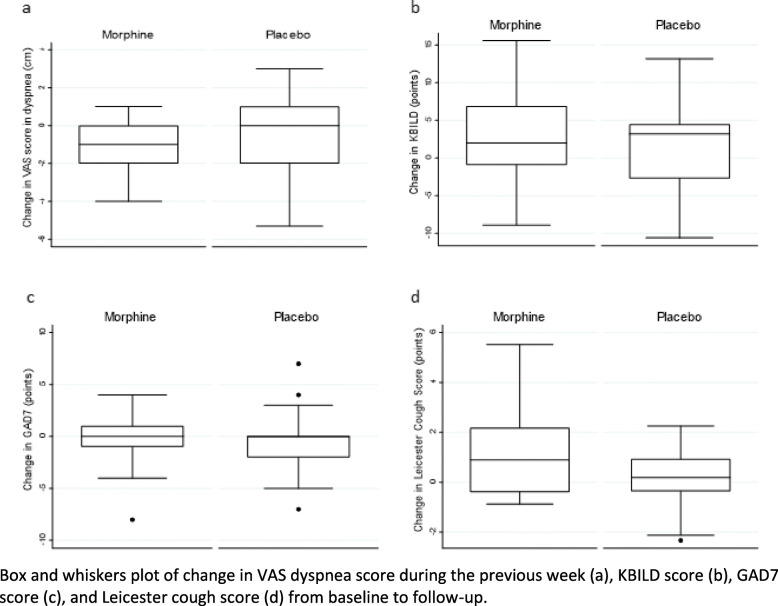
Changes in VAS dyspnea score during the previous week and questionnaires from baseline to follow-up. Box and whiskers plot of change in VAS dyspnea score during the previous week (**a**), KBILD score (**b**), GAD7 score (**c**), and Leicester cough score (**d**) from baseline to follow-up

The VAS dyspnea score during the previous week was reduced from baseline to follow up by 1.1 ± 0.33 cm in the morphine group (*P* < 0.01), whereas it was reduced by 0.35 ± 0.47 cm in the placebo group (*P* < 0.5). However, the reduction from baseline was not significantly different between the two groups (*p* = 0.2) (Table 2, Fig. 2 a). There was a statistically significant difference in the change from baseline to 1 h after medication intake in the Borg dyspnea score after the 6MWT and heart rate between the morphine and the placebo group, respectively. Furthermore, there was a statistically significant difference between groups concerning change of respiratory rate at follow-up compared to baseline. However, the differences were all due to a change in the placebo group; there were no changes in the morphine group (Table 2). There was no difference in the change of scores in the KBILD, the GAD7 and the Leicester cough score questionnaires between the two groups (Table 2, Fig. 2 b-d.)

**Table 2:** Primary and secondary study endpoints from baseline to follow-up after the first dose of medication and after 1 week of treatment with morphine or placebo

	Baseline		Change from baseline to 1 h after first dose of medication		Change from baseline to follow-up	
	Morphine	Placebo	Morphine	Placebo	Morphine	Placebo
VAS dyspnea during the last week (cm)	4.3 ± 0.37	4.7 ± 0.53			−1.1 ± 0.33†	−0.35 ± 0.46
VAS dyspnea score during the last hour (cm)	2 [1.6–4]	2.75 [0–6]	0 [− 1–0]	0 [− 3–0]	−1.2 ± 0.6	− 0.2 ± 0.7
VAS cough score during the last hour (cm)	0.5 [0–3]	0 [0–1.7]	0 [−1–0]	0[− 0.7–0] †	0 [− 2–0] †	0[− 0.5–0]
VAS cough score during the last week (cm)	1 [0–3]	1 [0–3]			−0.15 ± 0.24	−0.07 ± 0.34
6MWT distance (m)	338 ± 27	350 ± 30	12 ± 8	1 ± 8	10 ± 9	5 ± 6
PaO2 baseline (kPa)	11 ± 0.7	10 ± 0.7	−0.4 [− 3–0.5]	−0.05 [− 1.1–0.8]	−0.6 [− 1.3–0.9]	−0.2 [− 0.8–1]
PaCO2 baseline (kPa)	5.3 ± 0.3	4.8 ± 0.2	0.1 [−0.1–0.3]	0 [− 0.3–0.05]	0.2 ± 0.1	0.02 ± 0.1
Peripheral saturation (%)	96 ± 0.6	96 ± 0 .7	−1.5 ± 0.7†	−3.1 ± 0.7†	−2 [− 5–1]	−1 [− 2–1]
Borg score before 6MWT	1.8 ± 0.4	2.0 ± 0.6	− 0.1 ± 0.4	−0.2 ± 0.2	−0.7 ± 0.5	−0.2 ± 0.44
Borg score after 6MWT	6.5 ± 0.4	6.7 ± 0.5	0 [0–0]	0 [0–1]*	0.4 ± 0.6	−0.03 ± 0.5
Desaturation during 6MWT (%-points)	−11.5 ± 1.2	−13.0 ± 1.7	0.6 ± 0.6	1.3 ± 1.5	−3 ± 0.8†	− 0.4 ± 1.3
Systolic BP (mmHg)	136 ± 4	134 ± 4	4.6 ± 4.3	−1.0 ± 3.4	1.2 ± 4.9	0.1 ± 2.9
Respiratory rate (/min)	22 ± 1.3	22 ± 1.7	0 [0–2]	2 [0–3]	− 0.8 ± 1.1	2.6 ± 1.1* †
Heart rate (/min)	78 ± 4.0	80 ± 2.5	−1 [− 8–5]	− 6 [− 10--4]*†	−2.1 ± 2.5	−5.2 ± 2.7
FVC (l)	2.0 ± 0.1	2.0 ± 0.1	−0.06 ± 0.03	−0.1 ± 0.06†	−0.1 ± 0.03†	−0.2 ± 0.05†
KBILD score	51 ± 1.7	49 ± 2.9			2.9 ± 1.6	1.6 ± 1.5
Leicester cough score	17 [12–19]	18 [14–20]			1.2 ± 0.4 †	0.2 ± 0.3
GAD7 score	1 [0–5]	0.5 [0–3]			−0.4 ± 0.7	−0.6 ± 0.8

*VAS* visual analog scale, *6MWT* 6 min walk test, *PaO2* partial oxygen pressure; mmHg: millimeters of mercury, *BP* blood pressure, *FVC* forced vital capacity, *KBILD* Kings brief interstitial lung disease, *GAD7* general anxiety and depression

* Change from baseline significantly different from placebo group (*p* < 0.05); † *p* < 0.05 compared to baseline value within group

### Adverse effects

Constipation, nausea and confusion were reported significantly more often at follow-up than at baseline in the morphine group, but not in the placebo group (Table 3).

**Table 3:** Reported symptoms and side effects from baseline to follow-up after 1 week of treatment with morphine or placebo

	Morphine group		Placebo group	
Side effect	Baseline	Follow-up	Baseline	Follow-up
Constipation	2	7*	2	2
Nausea	7	8*	3	4
Headache	10	5	4	5
Dizziness	2	12	8	8
Confusion	8	7*	3	2
Other	0	7 (1 vomiting)	10	10

* *P*-value for change in proportion from baseline to follow-up < 0.05 vs. placebo

## Discussion

To our knowledge, this is the first prospective placebo-controlled randomised intervention study of morphine treatment for chronic breathlessness in patients with fILD. The main findings were that 5 mg morphine, four times a day was not effective in reducing dyspnea measured on a VAS scale. There was a trend for improved dyspnea in the intervention group, but this was not statistically significant when compared to the placebo group. Furthermore, we found no other effects of morphine based on measurements after the first dose and after 1 week. A few statistically significant differences in changes from baseline to measurement of endpoints were found between groups, but these were explained by changes in the placebo group. On the other hand, we found no indications of respiratory depression as only well-known adverse effects such as constipation, nausea and confusion were reported more often in the morphine group. Only one patient in the placebo group stopped the treatment due to a worsening of breathlessness.

Only a few studies on the effect of opioids in fILD patients have previously been conducted (8–10). Allen et al [9] did a case series study of 11 elderly patients with IPF who were injected with 2.5 mg of diamorphine subcutaneously. Although it was not a randomised placebo-controlled trial, the results seemed promising as they had a subjective, sustained positive effect on breathlessness. These results were seconded by two retrospective studies from Japan investigating continuous intravenous or subcutaneous administration of morphine in terminally ill patients with fILD, which reported relief of breathlessness in the majority of patients [8, 10]. Our finding that a daily dose of 20 mg of oral morphine had no statistically significant effect on dyspnea or other endpoints in fILD patients is in contrast with this and two other studies in mainly COPD patients that also suggested a positive effect of opioids [13, 14]. However, only the study by Abernethy was a randomized placebo-controlled study. They included 38 patients, and used 20 mg sustained release morphine sulphate per day for 4 days, and reported improvements in dyspnea during morning and evening measured on a 100 mm VAS scale [14].

However, our results concur with two recent large randomised placebo-controlled trials in mainly COPD patients with dyspnea [15, 16]. Currow et al investigated the effect of 20 mg sustained release oral morphine sulphate for 1 week in 284 patients, while Ferreira et al. investigated 4 weeks of 15 mg sustained release oxycodone per day in 157 patients. None of the studies found an effect of the opioid on “breathlessness now” measured on a VAS scale or on other endpoints. Furthermore, a number of other small-scale studies investigating the effect of opioids on chronic breathlessness primarily in patients with COPD did not point towards an effect [17–24].

Thus, even though a controlled study mainly in COPD patients, and some uncontrolled studies in fILD patients support the use of morphine for ease of chronic breathlessness, our results question the use in fILD, and is supported by recent results from patients with chronic breathlessness due to other aetiologies. Ander et al. [12] showed that a change in VAS dyspnea score of 2.1 cm was clinically relevant in a group of patients with heart failure that presented to the emergency department due to worsening of breathlessness. When reading the study of Johnson et al. [25], one can question if the clinically important difference is less than the chosen 21 mm. They estimated that a reduction in VAS score of 9 mm represents a clinically detectable change and our study might therefore be underpowered. Whether our and others’ negative results are influenced by dose and the chosen endpoints is not evident. When we planned our study, we chose the dose based on the previous study showing an effect [14]. Furthermore, a previous study in patients with chronic breathlessness mainly due to COPD or cancer, Currow et al. found that a 10 mg sustained oral morphine was safe and effective in 70% of responders to the treatment [13]. Therefore, we consider our choice of dose reasonable. We chose morphine drops as it is used in our daily clinical practice and often used in palliative treatment of patients with breathlessness. There is no evidence supporting this formulation of drug administration in patients with fILD, and one could speculate if the results would have been different if a slow-release formulation of morphine had been used. However, the above-mentioned recent studies speak against this, although they were mainly conducted in non-fILD patients. In theory, an effective treatment against chronic breathlessness could result in the patients pushing the intensity of their physical activities to the same level of breathlessness as before, and therefore a change in VAS would not necessarily be detected. But if this was so, we would expect a significant increase in 6MWT distance as a measure of their increased capacity, and this was not the case. So therefore, the chosen endpoint is not expected to explain the lack of effect in our study.

Safety concerns that opioids may cause respiratory depression, frequent hospitalisation and even premature death have for some been a barrier to treat patients with fILD with morphine. Bajwah et al. showed [26] that treatment with neither low- or high-dose opioids was associated with increased hospitalisation nor mortality in a population-based longitudinal cohort study in patients with fILD. Although our study period was only 1 week, the treatment with morphine drops was safe and only expected side effects were observed.

The strength of this study is that it is a randomised, placebo-controlled study design evaluating both objective and qualitative endpoints solely in fILD patients. The qualitative aspect is important when dealing with palliation. However, our study also has some limitations. The short duration of the trial might have affected the lack of changes in the questionnaires that includes questions concerning a time period of up to 2 weeks in retrospect. Also, daily measurements of symptomatic benefits and harms could add to the detection of changes and symptom burden. In spite of the power calculation and due to the heterogeneity of interstitial lung diseases, inclusion of a larger population may be necessary to show an effect of opioids.

Patients with fILD have a high symptom burden as their disease progresses and need palliative treatment for their symptoms [27]. Shortness of breath [6] can be excruciating and affect daily activities and quality of life. As there is no current medication approved for the treatment of chronic breathlessness, treatment modalities to relieve breathlessness are warranted. Our study does not support the use of morphine in this setting. However, it cannot be excluded that larger and longer-lasting randomised placebo-controlled studies investigating treatment of chronic breathlessness with opioids in flexible doses could reveal an effect.

## Conclusion

In conclusion, oral morphine drops of 5 mg four times a day in patients with fILD did not significantly reduce dyspnea VAS score in 1 week compared to placebo. The treatment seems safe as it did not induce respiratory depression and there no severe adverse events were seen. As expected, treatment with morphine was related to an increased risk of constipation, nausea and confusion.

## Data Availability

All data, including study protocol, used during the study are available on reasonable request.

## Abbreviations

DLCO: diffusion capacity
fILD: fibrotic Interstitial lung disease
FVC: forced vital capacity
GAD-7: Generalized Anxiety Disorder-7 questionnaire
HRCT: high-resolution computer tomography
IPF: idiopathic pulmonary fibrosis
KBILD: King’s Brief Interstitial Lung Disease health status questionnaire
MRC: Medical Research Council score
PaO_2_: arterial partial oxygen pressure
VAS: visual analogue score
6MWT: six-minute walk test

## References

[1] Hyldgaard C, Hilberg O, Muller A, Bendstrup E (2014). A cohort study of interstitial lung diseases in Central Denmark. Respir Med.

[2] Kolb M, Vašáková M (2019). The natural history of progressive fibrosing interstitial lung diseases. Respir Res.

[3] Richeldi L, du Bois RM, Raghu G, Azuma A, Brown KK, Costabel U (2014). Efficacy and safety of Nintedanib in idiopathic pulmonary fibrosis. N Engl J Med.

[4] King TE, Bradford WZ, Castro-Bernardini S, Fagan EA, Glaspole I, Glassberg MK (2014). A phase 3 trial of Pirfenidone in patients with idiopathic pulmonary fibrosis. N Engl J Med.

[5] Dempsey TM, Sangaralingham LR, Yao X, Sanghavi D, LA Shah ND (2019). Clinical effectiveness of Antifibrotic medications for idiopathic pulmonary fibrosis. Am J Respir Crit Care Med.

[6] Kreuter M, Bendstrup E, Russell AM, Bajwah S, Lindell KAY (2017). Palliative care in interstitial lung disease: living well. Lancet Respir Med.

[7] Glaspole IN, Chapman SA, Cooper WA, Ellis SJ, Goh NSHP (2017). Health-related quality of life in idiopathic pulmonary fibrosis: data from the Australian IPF registry. Respirology..

[8] Takeyasu M, Miyamoto A, Kat D, Takahashi Y, Ogawa KMK (2016). Continuous intravenous morphine infusion for severe dyspnea in terminally ill interstitial pneumonia patients. Intern Med.

[9] Allen S, Raut S, Woollard JVM (2005). Low dose diamorphine reduces breathlessness without causing a fall in oxygen saturation in elderly patients with end-stage idiopathic pulmonary fibrosis. Palliat Med.

[10] Matsuda Y, Maeda I, Tachibana K, Nakao K, Sasaki YSC (2017). Low-dose morphine for dyspnea in terminally ill patients with idiopathic interstitial pneumonias. J Palliat Med.

[11] Travis WD, Costabel U, Hansell DM, King TE, Lynch DA (2013). An official American Thoracic Society/European Respiratory Society statement: update of the international multidisciplinary classification of the idiopathic interstitial pneumonias. Am J Respir Crit Care Med.

[12] Ander DS, Aisiku IP, Ratcliff JJ, Todd KH, Gotsch K. Measuring the dyspnea of decompensated heart failure with a visual analog scale: how much improvement is meaningful? Congest Heart Fail. 2004;10(4):188-91. 10.1111/j.1527-5299.2004.03475.x 15314477

[13] Currow DC, McDonald C, Oaten S, Kenny B, Allcroft PFP (2011). Once-daily opioids for chronic dyspnea: a dose increment and pharmacovigilance study. J Pain Symptom Manag.

[14] Abernethy AP, Currow DC, Frith P, Fazekas BS, McHugh ABC (2003). Randomised, double blind, placebo controlled crossover trial of sustained release morphine for the management of refractory dyspnea. Br Med J.

[15] Currow D, Louw S, McCloud P, Fazekas B, Plummer J (2020). Regular, sustained-release morphine for chronic breathlessness: a multicentre, couble-blind, randomised, placebo-controlled trial. Thorax..

[16] Ferreira DH, Louw S, McCloud P, Fazekas B, McDonald CF et al. Controlled-release oxycodone versus placebo in the treatment of chronic breathlessness- a multi-site randomised placebo controlled trial. J Pain Symptom Manage. 2019;59(3):581-9. 10.1016/j.jpainsymman.2019.10.017 31655189

[17] Jankelson D, Hosseini K, Mather LE, Seale JPYI (1997). Lack of effect of high doses of inhaled morphine on exercise endurance in chronic obstructive pulmonary disease. Eur Respir J.

[18] Leung R, Hill PBJ (1996). Effect of inhaled morphine on the development of breathlessness during exercise in patients with chronic lung disease. Thorax..

[19] Young IH, Daviskas EKV (1989). Effect of low dose nebulised morphine on exercise endurance in patients with chronic lung disease. Thorax..

[20] Poole PJ, Veale AGBP (1998). The effect of sustained-release morphine on breathlessness and quality of life in severe chronic obstructive pulmonary disease. Am J Respir Crit Care Med.

[21] Noseda A, Carpiaux JP, Markstein C, Meyvaert A (1997). de M V. disabling dyspnoea in patients with advanced disease: lack of effect of nebulized morphine. Eur Respiriratory J.

[22] Light RW, Stansbury DWWJ (1996). Effect of 30 mg of morphine alone or with promethazine or prochlorperazine on the exercise capacity of patients with COPD. Chest..

[23] Eiser N, Denman WT, West CLP (1991). Oral diamorphine: lack of effect on dyspnoea and exercise tolerance in the “pink puffer” syndrome. Eur Respir J.

[24] Jensen D, Alsuhail A, Viola R, Dudgeon DJ, DE Webb KAO (2012). Inhaled fentanyl citrate improves exercise endurance during high-intensity constant work rate cycle exercise in chronic obstructive pulmonary disease. J Pain Symptom Manag.

[25] Johnson MJ, Bland JM, Oxberry SG, Abernethy AP, Currow DC (2013). Clinically important differences in the intensity of chronic refractory breathlessness. J Pain Symptom Manag.

[26] Bajwah S, Davies JM, Tanash H, Currow DC, Oluyase AOEM (2018). Safety of benzodiazepines and opioids in interstitial lung disease: a national prospective study. Eur Respir J.

[27] Ahmadi Z, Wysham NG, Lundström S, Janson C, Currow DCEM (2016). End-of-life care in oxygen-dependent ILD compared with lung cancer: a national population-based study. Thorax..

